# Stomach pullup for burnt out esophagus, an experience of 44 cases

**DOI:** 10.1186/1749-8090-10-S1-A14

**Published:** 2015-12-16

**Authors:** Amer Bilal

**Affiliations:** 1 Dept of Cardiothoracic Surgery, Lady Reading Hospital, Peshawar, Pakistan

## Background/Introduction

Gastric pull-up with cervical anastomosis is a safe procedure, which can be performed for the treatment of non-dilatable corrosive esophageal stricture.

## Aims/Objectives

To determine the outcome of surgical management of Non-dilatable corrosive esophageal stricture.

## Method

Computerized clinical records of forty four diagnosed corrosive esophageal stricture patients from March 2007 to Dec 2014 were retrospectively analyzed. Patient of all ages, both sexes, medically fit and corrosive stricture involving thoracic esophagus only were included in the study. Medically unfit patients and corrosive stricture involving the larynx, cervical esophagus and stomach were excluded from the study. Patients were registered through OPD. After necessary preoperative workup the patients were subjected to the surgical procedure.

## Results

Out of 44 patients, 28 were male and 16 were female. Age ranges from 6 to 65 years with a median age of 21 years. Accidental ingestion was observed in 31 patients and Suicidal in 13. Acidic injury was specified in 19 patients whereas caustic ingestion n 25 patients. Average time between chemical injury and surgery was 4 weeks. In all cases we did esophagectomy and stomach was used as a conduit with gastroesophageal anastomosis in the neck. Morbidity was 3 (6.81%) including anastomotic leak in one and anastomotic stricture in two. Overall mortality rate was 2 (4.54%) due to respiratory failure.

## Discussion/Conclusion

Good and satisfactory results can be obtained in 90% of the patients after gastroesophagoplasty for Non-dilatable corrosive esophageal stricture.

